# The efficacy of interventions to prevent type 2 diabetes among women with recent gestational diabetes mellitus—A living systematic review and meta‐analysis

**DOI:** 10.1111/1753-0407.13590

**Published:** 2024-08-13

**Authors:** Vivian Y. Lee, Mohammad R. Monjur, Joseph Alvin Santos, Anushka Patel, Rong Liu, Gian Luca Di Tanna, Yashdeep Gupta, Alpesh Goyal, Saumiyah Ajanthan, Devarsetty Praveen, J. K. Lakshmi, H. Asita de Silva, Nikhil Tandon

**Affiliations:** ^1^ The George Institute for Global Health Sydney New South Wales Australia; ^2^ Faculty of Medicine University of New South Wales Kensington New South Wales Australia; ^3^ South Eastern Sydney Local Health District Sydney New South Wales Australia; ^4^ Department of Innovative Technologies University of Applied Sciences and Arts of Southern Switzerland Manno Switzerland; ^5^ Department of Endocrinology and Metabolism All India Institute of Medical Sciences New Delhi India; ^6^ RemediumOne Colombo Sri Lanka; ^7^ The George Institute for Global Health New Delhi India; ^8^ Prasanna School of Public Health Manipal India; ^9^ Clinical Trials Unit, Department of Pharmacology, Faculty of Medicine University of Kelaniya Colombo Sri Lanka

**Keywords:** impaired glucose tolerance, postpartum, prevention

## Abstract

**Background:**

While previously considered a transient condition, with no lasting adverse impact, gestational diabetes mellitus (GDM) is now a well‐established risk factor for developing type 2 diabetes mellitus (T2DM). The risk of developing T2DM appears to be particularly high in the first few years after childbirth, providing a compelling case for early intervention. This review provides an up‐to‐date systematic review and meta‐analysis to assess the effectiveness of interventions to reduce incidence of T2DM in women with a recent history of GDM.

**Methods:**

The search was conducted on October 20, 2023 with an annual surveillance planned for the next 5 years to maintain a living systematic review. The inclusion criteria were randomized controlled trials of any type in women within 5 years of GDM‐complicated pregnancy that reported outcomes of T2DM diagnosis or measures of dysglycemia with a follow‐up of at least 12 months.

**Results:**

Seventeen studies met our inclusion criteria and have been included in this review. There were 3 pharmacological and 14 lifestyle interventions. Intervention was not associated with significant reduction in the primary outcome of T2DM (risk ratio, 0.78; 95% confidence interval [CI]: 0.43–1.41; *p* = 0.41; *I* ^2^ = 79%) compared with the control group (placebo or usual care). However, meta‐analysis of the four studies reporting hazard ratios suggested a reduction in diabetes incidence (hazard ratio, 0.68; 95% CI: 0.48–0.97; *p* = 0.03; *I* ^2^ = 31%).

**Conclusion:**

This review provides equivocal evidence about the efficacy of interventions to reduce the risk of T2DM in women within 5 years of GDM‐complicated pregnancy and highlights the need for further studies, including pharmacotherapy.

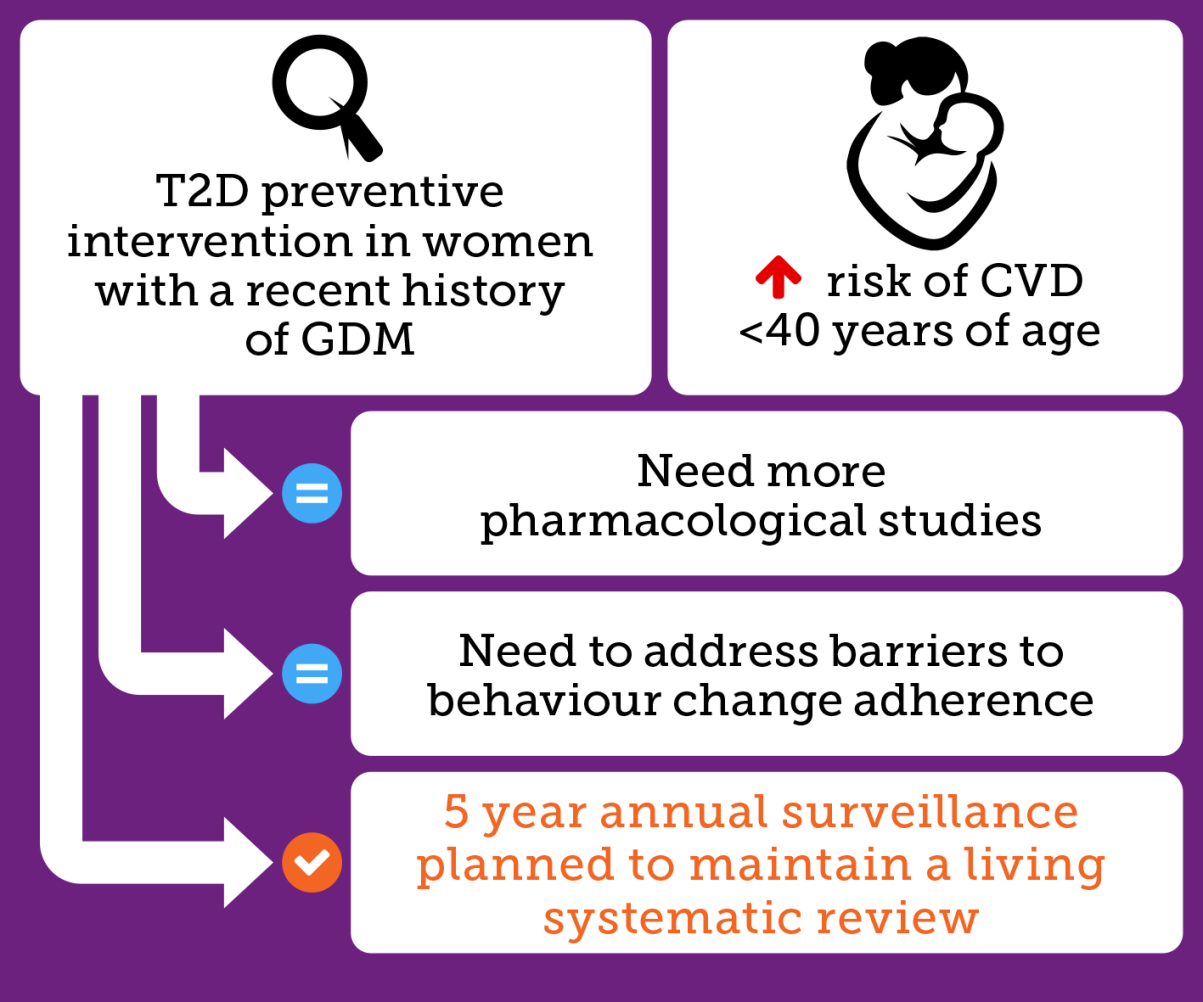

## Graphical abstract

- GDM is now a well‐established risk factor for developing T2DM. This risk appears to be particularly high in the first few years after childbirth, providing a compelling case for early intervention.
- This review found equivocal evidence about the efficacy of preventive interventions on development of T2DM in women with a recent (within 5 years) history of GDM.
- Very few data evaluating pharmacotherapy were available, highlighting the need for further studies.

## 1 INTRODUCTION

Diabetes is a leading and growing cause of morbidity and mortality in adults worldwide.^1^ An estimated 537 million adults live with diabetes, with this condition accounting for approximately 6.7 million deaths in 2021.^2^ Importantly, diabetes‐related disability adjusted life‐year loss, contributing to the largest rise in global burden among noncommunicable diseases, increasing by 148% from 1990 to 2019.^3^ In response, the World Health Organization (WHO) launched the Global Diabetes Compact in 2021 aimed at sustaining improvements in diabetes prevention and care, through collaborative multidisciplinary initiatives with national and international bodies.^4^ Type 2 diabetes mellitus (T2DM) accounts for over 90% of cases and can be prevented or delayed.^2^ Major clinical trials and reviews have established the effectiveness of diet and lifestyle modifications, and of metformin, in preventing T2DM among certain older, high‐risk population groups.^5^

While previously considered a transient condition, with no lasting adverse impact, gestational diabetes mellitus (GDM) is now a well‐established risk factor for developing T2DM. In a most recent pooled analysis of 1.3 million women, women with prior GDM had a 10‐fold risk of developing T2DM compared with women without GDM, identifying a key high‐risk group.^6^ The prevalence of GDM in pregnancies varies markedly worldwide, ranging from 2% to 25%.^7^ Importantly, the risk of developing T2DM appears to be particularly high in the first few years after childbirth,^6^, ^8^ and those diagnosed with diabetes below 40 years of age are found to have an increased risk of cardiovascular disease than those diagnosed later, which provides a compelling case for early intervention.^9^

A recent systematic review of randomized trials in lifestyle interventions found benefits in reducing the incidence of T2DM among 3745 participants.^10^ No previous systematic reviews have been undertaken of pharmacotherapeutic approaches, or of all intervention types, to examine the effects on diabetes prevention in women with recent GDM. However, a scoping review of pharmacological interventions in women with a history of GDM suggested metformin to be effective with limited interpretability of other drugs (thiazolidinediones, troglitazone, pioglitazone, and vildagliptin).^11^ Therefore, we undertook a systematic review and meta‐analysis to assess the effectiveness of preventive interventions, of any type, to reduce the incidence of T2DM and biochemical markers in women with a recent history of GDM. Further analysis was conducted to examine the effect of specific intervention types. As several trials are in progress,^12^, ^13^, ^14^, ^15^, ^16^ we propose to conduct annual surveillance for the next 5 years to maintain a living systematic review.

## 2 METHODS

This study was conducted according to the 2015 Preferred Reporting Items for Systematic Reviews and Meta‐Analysis Protocols (PRISMA‐P) statement. The study protocol was registered with the international database of prospectively registered systematic reviews in health and social care (PROSPERO—CRD42021279891). A detailed study protocol outlining the predefined eligibility criteria, search strategy, data analysis methods, and risk of bias assessment has been previously published.^17^

### 2.1 Search strategy and selection criteria

A systematic search for peer‐reviewed articles on the online databases of PubMed, EMBASE and Web of Science was performed on October 20, 2023. The full search strategy^17^ used a combination of keywords, and their variations, pertinent to our population, intervention, and outcomes of interest, including “gestational diabetes mellitus,” “lifestyle,” “exercise,” “physical activity,” “randomized controlled trials,” “diabetes mellitus,” and various terms related to pharmacotherapy (e.g., “metformin,” “thiazolidinediones,” “dipeptidyl peptidase 4 inhibitors,” “sodium glucose transporter 2,” “dipeptidyl peptidase IV,” “liraglutide,” “hypoglycemic agents,” “hypoglycemic agents,” “antidiabetic,” “dietary supplement,” or “myo‐inositol”).

The eligibility criteria for inclusion were as follows: (1) Evaluation: randomized controlled trials; (2) Population: women within 5 years of a pregnancy complicated by GDM; (3) Intervention and comparator: any type including lifestyle, behavioral, psychological, or pharmacotherapy compared with another active intervention, usual care, or placebo; (4) Outcomes: diagnosis of T2DM or a measure of dysglycemia; (5) Follow‐up: minimum of 12 months. GDM was defined according to any recognized diagnostic criteria or based on medical record documentation. Where multiple publications of the same trial were present, the one with the longest available follow‐up period was included. There were no language restrictions. The reference lists of eligible articles and previous reviews were also screened for relevant studies. The living status of the systematic review will be maintained for 5 years after the protocol publication (November 2021),^17^ with an updated search in November 2024 and planned every 12 months thereafter.

### 2.2 Screening and data extraction

Screening and data extraction were conducted independently by two authors (VL and MRM), and any disagreements were resolved by consensus with a third author (AP). The abstracts were screened against the inclusion and exclusion criteria, followed by the full text of potentially eligible trials. Data from eligible articles confirmed by the authors were extracted, risk‐of‐bias assessed, and template for intervention description and replication (TIDieR) checklist completed by VL and MRM. The non‐English publications were screened and extracted by a native speaker who is a part of the research team (RL). Data were extracted using a standardized electronic template. Details of the extracted variables are presented in Data S1.

### 2.3 Outcomes

The primary outcome was the incidence of T2DM, as defined by the individual study. Secondary outcomes included fasting plasma glucose levels, fasting insulin levels, HOMA‐IR score, 2‐h oral glucose tolerance test (OGTT), Haemoglobin A_1_c (HbA_1_c), body weight, body mass index, waist circumference, total cholesterol, high‐density lipoprotein (HDL) cholesterol, low‐density lipoprotein (LDL) cholesterol, and triglycerides.

### 2.4 Data analysis

The details of eligible studies were summarized. Study characteristics and available baseline participant data were described by measures of central tendency. Random‐effects meta‐analysis using the Hartung–Knapp–Sidik–Jonkman method were conducted to generate an overall effect estimate for each outcome measure. The pooled estimates of effect sizes were reported as risk ratios for binary outcomes and as mean differences for continuous outcomes. For the primary outcome of interest incidence of T2DM, the overall effect estimate was reported both as risk ratio (including studies that reported binary data) and as hazard ratio (including studies that reported hazard ratio and its measure of variability). Subgroup analyses were conducted according to (1) the type of intervention (*lifestyle* vs. *pharmacological intervention*); (2) average race‐specific baseline body mass index (BMI) (*obese* vs. *nonobese*)^18^; and (3) income classification of the study country per the World Bank (*high‐income* vs. *low‐ and middle‐income*). Sensitivity analyses, excluding high risk of bias studies,^14^, ^19^, ^20^ and a suspected outlier^20^ identified through leave‐one‐out meta‐analysis, were conducted to check the robustness of the primary analysis results.

For continuous outcomes, postintervention and change‐from‐baseline data were combined in a single meta‐analysis. Study‐level estimates were converted to a common scale or unit before pooling (for example, fasting plasma glucose was converted to mmol/L if reported in other units of measurement). Standard errors (SE) and confidence intervals (CI) were converted to standard deviations (SD) using the equations outlined in the Cochrane Handbook.^21^ Medians and interquartile ranges were converted to means and SD using the methods proposed by Wan et al.^22^ For cluster trials, clustering was taken into account by calculating the trial's effective sample size. An intraclass correlation coefficient of 0.01 was used to obtain the trial's design effect, and a common design effect was applied across treatment arms.

Quantitative heterogeneity was assessed by evaluating the proportion of variability due to heterogeneity through the *I* ^2^ statistic and by using the Cochran Q test of homogeneity. Presence of small‐study effects was assessed by visual inspection of the contour‐enhanced funnel plots and through regression‐based Egger test.

Analyses were based on reported data based on the intention‐to‐treat principle. No missing data were imputed. Analyses were conducted using Stata version 17 (StataCorp LLC, College Station, TX, USA).

### 2.5 Risk of bias assessment

A risk of bias assessment for each published trial was conducted in duplicate using the Cochrane risk‐of‐bias tool (RoB 2).^23^ Studies were classified into “low risk of bias,” “unclear risk of bias” and “high risk of bias” using the RoB 2 tool by assessing several domains including random allocation sequence, allocation sequence concealment, blinding, outcome assessment, missing data, and analysis methods.

### 2.6 Implementation assessment

The implementation of interventions was assessed using the TIDieR checklist and guide.^24^

## 3 RESULTS

**FIGURE 1:**
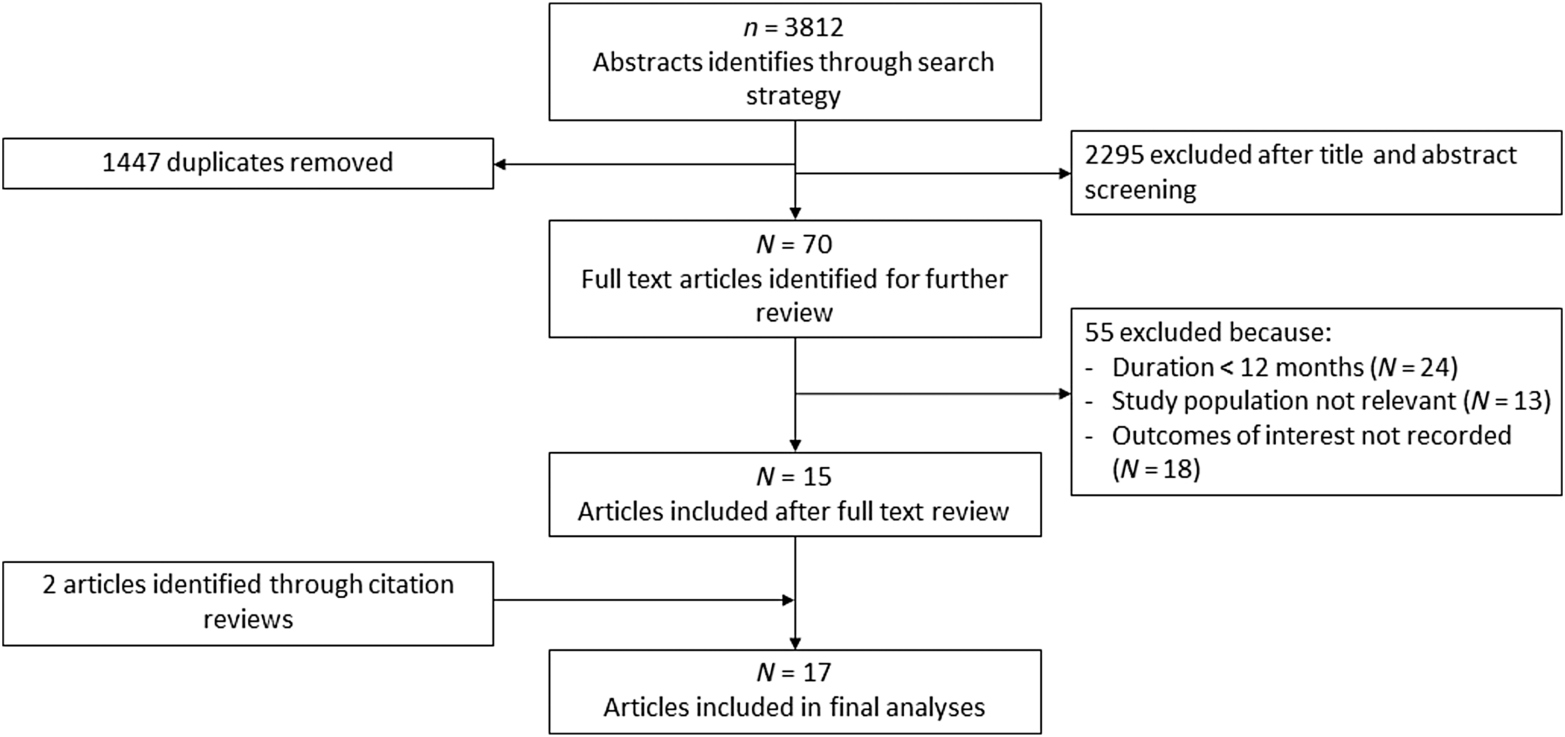
Preferred Reporting Items for Systematic Reviews and Meta‐Analysis (PRISMA) diagram.

A total of 3812 abstracts were identified by the search strategy, with 15 studies meeting the eligibility criteria. Two further articles were identified through manual citation searches, and no duplicate reports of interventions were included. A total of 17 studies were included in this review (Figure 1).

### 3.1 Study characteristics

The study characteristics, including description of the interventions and main reported findings on relevant outcomes, are presented in Table 1. Of the 17 studies, 16 studies were individual randomized controlled trials and one was a cluster randomized controlled trial.^25^ Eleven studies were conducted in high‐income countries,^20^, ^26^, ^27^, ^28^, ^29^, ^30^, ^31^, ^32^, ^33^, ^34^, ^35^ six studies in middle‐income countries,^19^, ^25^, ^36^, ^37^, ^38^, ^39^ and none in low‐income countries. Two studies were published in Chinese,^19^, ^39^ and 15 studies in English.^20^, ^25^, ^26^, ^27^, ^28^, ^29^, ^30^, ^31^, ^32^, ^33^, ^34^, ^35^, ^36^, ^37^, ^38^ Studies were reported between 1999 and 2022, with a mean number of 364 participants (range, 43–1601). The median intervention and follow‐up periods were both 24 months. The participants had a mean age of 34.5 years (range, 29.2 to 39.5 years) and a mean baseline body mass index of 28.9 kg/m^2^ (range, 23.8 to 37.2 kg/m^2^). Diagnosis of GDM in the participants was based on the WHO criteria in three,^25^, ^37^, ^38^ the Carpenter–Coustan criteria in five,^25^, ^28^, ^31^, ^34^, ^39^ the International Association of Diabetes and Pregnancy Study Group (IADPSG) diagnostic criteria in two,^26^, ^36^ the Australasian Diabetes in Pregnancy Society criteria in two,^29^, ^35^ the American Diabetes Association (ADA) criteria in one,^19^ medical records in one,^33^ and not reported in three.^20^, ^30^, ^32^

**TABLE 1:** Characteristics of eligible studies and participants.

Study (country and year)	Study description	Total sample size	Diagnosis criteria for GDM and glycemic status	Intervention period (months)	Follow‐up period (months)	Intervention type	Mean age at baseline (years)	Mean body mass index at baseline (kg/m^2^)
Wein et al. (Australia, 1999)^33^	Individual RCT of a simple versus intensified dietary modification among women with impaired glucose tolerance during 2‐yearly post‐GDM screening visits. Not clear that all women were randomized within 5 years of GDM‐affected pregnancy. Primary outcome was T2DM.	200	GDM: Hospital records Glycemic status: WHO criteria (1980)	51 (median)	51 (median)	Diet and physical activity	38.7	25.4
Buchanan et al. (USA, 2002)^31^	Individual double‐blind RCT of troglitazone versus placebo among Hispanic women with GDM within the previous 4 years and high‐risk of diabetes based on previous blood glucose levels. Primary outcome was T2DM with treatment and follow‐up discontinued prematurely due to drug withdrawal from market. Approximately 40% of participants attended their final visit a median of ~8 months after cessation of study drug.	266	GDM: Carpenter–Coustan criteria Glycemic status: WHO criteria (1980)	53 (median)	>53 (median)	Pharmacotherapy Troglitazone	34.6	30.4
Cheung et al. (Australia, 2011)^35^	Individual RCT of a lifestyle intervention on improving physical activity versus general lifestyle information in women who had GDM in the last 4 years. Primary outcome was change in physical activity. The intervention focused on patient‐centered counseling and self‐management education techniques to change behavior.	43	GDM: ADIPS criteria Glycemic status: n/a	12	12	Physical activity	36.5	27.2
Hu et al. (China, 2012)^38^	Individual RCT of an individualized lifestyle intervention focused on diet and physical activity versus general lifestyle information. Women had GDM between 2005 and 2009 and were randomized between 2009 and 2011; thus, some participants may have experienced GDM >5 years previously. Primary outcome was T2DM.	1180	GDM: WHO criteria (1999) Glycemic status: WHO criteria (1999)	24	24	Diet and physical activity	32.4	23.9
Yu et al. (China, 2012)^19^	Individual RCT of a lifestyle intervention focused on diet and physical activity versus “no treatment” in women who had GDM. Women were recruited in 2009 and hence had GDM <5 years but the exact time was not stated. Outcomes were body mass index, insulin resistance index, islet beta cell function index, and insulin secretion index.	126	GDM: ADA criteria (1997) Glycemic status: WHO criteria (1999)	24	24	Diet and physical activity	29.6	25.1
Shek et al. (Hong Kong, China, 2013)^37^	Individual RCT of a lifestyle intervention focused on diet and physical activity versus “no treatment” in women not treated with insulin during their index GDM‐complicated pregnancy. Women were randomized at 6 weeks postnatal screening if found to have impaired glucose tolerance. Primary outcome was T2DM.	450	GDM: WHO criteria (1999) Glycemic status: WHO criteria (1999)	36	36	Diet and physical activity	39.0	25.4
Guo et al. (China, 2013)^39^	Individual RCT of a lifestyle intervention focused on diet and physical activity versus “no treatment” in women who had GDM. Women were recruited between January 2010 and January 2011 and hence had GDM <5 years, but the exact time not stated. Outcomes were incidence of postpartum depression, T2DM, health literacy score, and glucose concentration.	100	GDM: Carpenter–Coustan criteria Glycemic status: ADA criteria (2010)	20	20	Diet and physical activity	28.2	*NR*
Nicklas et al. (USA, 2014)^34^	Individual RCT of a web‐based lifestyle intervention modification program (Balance after Baby) focused on diet and physical activity versus general lifestyle information. Women were randomized 6 weeks postpartum. Primary outcome was changes in body weight.	75	GDM: Carpenter–Coustan criteria or by medical record‐documented clinician diagnosis. Glycemic status: n/a	12	12	Diet and physical activity	33.5	31.4
O'Dea et al. (Ireland 2015)^26^	Individual RCT of an intensive individualized lifestyle intervention versus standard health care advice among women with GDM in the previous 1–3 years and either impaired fasting glucose, impaired glucose tolerance or insulin resistance with additional risk factors. The primary outcome was change in fasting glucose levels from baseline to 1 year follow‐up.	50	GDM: IADPSG criteria Glycemic status: n/a	3	12	Diet and physical activity	*NR*	35.5
Perez‐Ferre et al. (Spain, 2015)^27^	Individual RCT of a nutrition and supervised exercise lifestyle intervention versus group education and usual care among women with GDM and without impaired fasting glucose at their first 7–12 weeks postpartum evaluation. The total period of intervention is unclear. The primary outcome was dysglycemia (impaired fasting glucose and/or impaired glucose tolerance, or T2DM).	260	GDM: Carpenter–Coustan criteria Glycemic status: n/a	6	36	Diet and physical activity	35.0	26.4
O'Reilly et al. (Australia, 2016)^29^	Individual RCT of an individual and group lifestyle program versus usual care among women with recent GDM (median 8 months postdelivery). The primary outcomes were changes in fasting glucose, waist circumference, and weight at 12 months.	573	GDM: ADIPS criteria Glycemia status: n/a	12	12	Diet and physical activity	33.8	28.8
Hummel et al. (Germany, 2018)^28^	Individual double‐blind RCT of vildagliptin versus placebo among women with insulin‐treated GDM within the previous 9 months. The primary outcome was worsening glycemia from baseline (development of new impaired glucose tolerance, impaired fasting glucose, or T2DM).	113	GDM: Carpenter–Coustan criteria Glycemic status: ADA criteria (1997)	~26 (median)	~36 (median)	Pharmacotherapy Vildagliptin	34.5	28.2
McManus et al. (Canada, 2018)^32^	Individual RCT of a personalized lifestyle intervention involving close family members versus standard advice among women with recent GDM, recruited during pregnancy with a predicted postpartum body mass index of 24 kg/m^2^ or greater (mean 8.5 months postdelivery). The primary outcome was body weight at 12 months.	170	GDM: n/a Glycemic status: n/a	12	12	Diet and physical activity	34.1	35.2
Zilberman‐Kravitz et al. (Israel, 2018)^20^	Individual RCT of dietary counseling and guided physical activity intervention versus standard information (4:3 allocation) among women with recent GDM contacted 3–4 months postdelivery. The intervention comprised individual and group sessions over an uncertain period of time. The primary outcome was HOMA‐IR at 1 and 2 years.	180	GDM: n/a Glycemic status: n/a	*21*	24	Diet and physical activity	35.7	29.8
Elkind‐Hirsch et al. (USA, 2020)^30^	Individual double‐blind RCT of metformin + liraglutide versus metformin + placebo among women with GDM within 12 months, BMI ≥25 kg/m^2^, and abnormal oral glucose tolerance test. The primary outcome was change in insulin secretion‐sensitivity index.	153	GDM: n/a Glycemic status: ADA criteria	21	21	Pharmacotherapy Metformin Liraglutide	*NR*	35.5
Tandon et al. (India, Sri Lanka, Bangladesh, 2022)^36^	Individual RCT of a primarily group‐based lifestyle intervention among women with recent GDM (median 6.5 months postdelivery). The primary outcome was worsening glycemia from baseline (development of new impaired glucose tolerance, impaired fasting glucose, or T2DM).	1601	GDM: IADPSG criteria (2016) Glycemic status: ADA criteria (2018)	12	14 (median)	Diet and physical activity	30.9	26.6
Lee et al. (Malaysia, 2022)^25^	Cluster RCT of a personalized lifestyle intervention focused around lifestyle modifications and emphasis on the importance of diabetes screening versus general lifestyle information. The intervention commenced from 36 weeks of gestation. The primary outcome was progression to T2DM after 2 years of study period.	650	GDM: WHO criteria (2013) Glycemic status: WHO 2013 with HbA_1_c ≥6.3% rather than ≥6.5%	24	24	Diet and physical activity	31.6	27.2

*Note*: ~ denotes proposed follow‐up period, which changed if individuals were diagnosed with diabetes.

Abbreviations: ADA, American Diabetes Association; ADIPS, Australasian Diabetes in Pregnancy Society; GDM, gestational diabetes mellitus; IADPSG, International Association of Diabetes and Pregnancy Study Groups; n/a, not avai; T2DM, type 2 diabetes mellitus; WHO, World Health Organization; HbA_1_c, Haemoglobin A_1_c .

Three studies were pharmacological interventions,^28^, ^30^, ^31^ while 14 were lifestyle interventions.^19^, ^20^, ^25^, ^29^, ^32^, ^33^, ^34^, ^35^, ^36^, ^37^, ^38^, ^39^ The pharmacological interventions examined the effect of troglitazone^31^ and vildagliptin^28^ against placebo, and a combination of metformin and liraglutide against a combination of metformin and placebo.^30^ Thirteen lifestyle interventions aimed to improve diet and physical activity, and one intervention aimed to increase physical activity alone.^35^ All intervention groups were compared with usual care. Lifestyle interventions were delivered face to face in six,^20^, ^25^, ^26^, ^27^, ^32^, ^37^ virtually via telephone or web‐based in two,^33^, ^34^ and using a combination of approaches in six studies.^19^, ^29^, ^35^, ^36^, ^38^, ^39^ The participants were individually contacted and supported in seven studies,^19^, ^25^, ^33^, ^34^, ^35^, ^37^, ^39^ and seven studies provided both individual and group support.^20^, ^26^, ^27^, ^29^, ^32^, ^36^, ^38^ Lifestyle intervention began during pregnancy in two studies,^25^, ^32^ and postpartum in 12 studies (range, 6–200+ weeks postpartum).^26^, ^27^, ^29^, ^32^, ^33^, ^34^, ^35^, ^36^, ^38^, ^39^ Eight studies^19^, ^25^, ^26^, ^27^, ^34^, ^37^, ^38^, ^39^ provided individualized dietary guidance while five provided general guidance.^20^, ^29^, ^32^, ^33^, ^36^ Four studies provided individualized dietary support with a dietitian,^26^, ^33^, ^37^, ^38^ and nine studies provided a group session with a dietitian or someone equivalent.^19^, ^20^, ^25^, ^27^, ^29^, ^32^, ^34^, ^36^, ^39^ Seven studies^25^, ^26^, ^27^, ^34^, ^35^, ^37^, ^38^ provided individualized physical activity guidance while seven did not.^19^, ^20^, ^29^, ^32^, ^33^, ^36^, ^39^ Two studies provided supervised exercise sessions^26^, ^27^ and 12 studies did not. Two studies reported adherence to intervention, where one reported 34% attended at least one walking group,^27^ and the other reported 58.3% attended 50% or more of the sessions.^33^ The retention rate was 100% in two studies,^19^, ^39^ over 90% in four studies,^27^, ^33^, ^34^, ^38^ over 70% in five studies,^26^, ^29^, ^35^, ^36^, ^37^ and between 45.8% and 57.8% in three studies.^20^, ^25^, ^32^ The intervention study characteristics are summarized in a TIDieR table (Data S1).

### 3.2 Publication bias and quality

The risk‐of‐bias summary is provided in Figure S1. Overall, 53% (*n* = 9)^28^, ^29^, ^31^, ^32^, ^33^, ^34^, ^36^, ^37^, ^38^, ^39^ of the studies were rated as having a low risk of bias, 29% (*n* = 5)^25^, ^26^, ^27^, ^30^, ^35^ as having an unclear risk of bias, and 18% (*n* = 3)^25^, ^26^, ^27^, ^30^, ^35^ as having a high risk of bias. Two studies were rated as having a high risk of bias due to the insufficient information in the publications,^19^, ^39^ and one due to a significant number of dropouts at the final follow‐up (40% lost to follow‐up).^20^ Studies rated as having unclear risk of bias^25^, ^26^, ^27^, ^30^, ^35^ were mainly due to lack of information provided by the studies, especially of allocation sequence concealment (*n* = 1),^26^ missingness of the data (*n* = 3),^25^, ^30^, ^35^ and appropriate prespecified analysis plan (*n* = 1).^37^ The Egger test and the funnel plot for the 10 studies that report the incidence of T2DM suggest absence of publication bias (Figure S2; *p* = 0.38). The Egger test also suggests absence of publication bias for the secondary outcomes (*p* > 0.05) apart from BMI (*p* = 0.038), although the contour‐enhanced funnel plot looks symmetrical.

### 3.3 Outcomes

**FIGURE 2:**
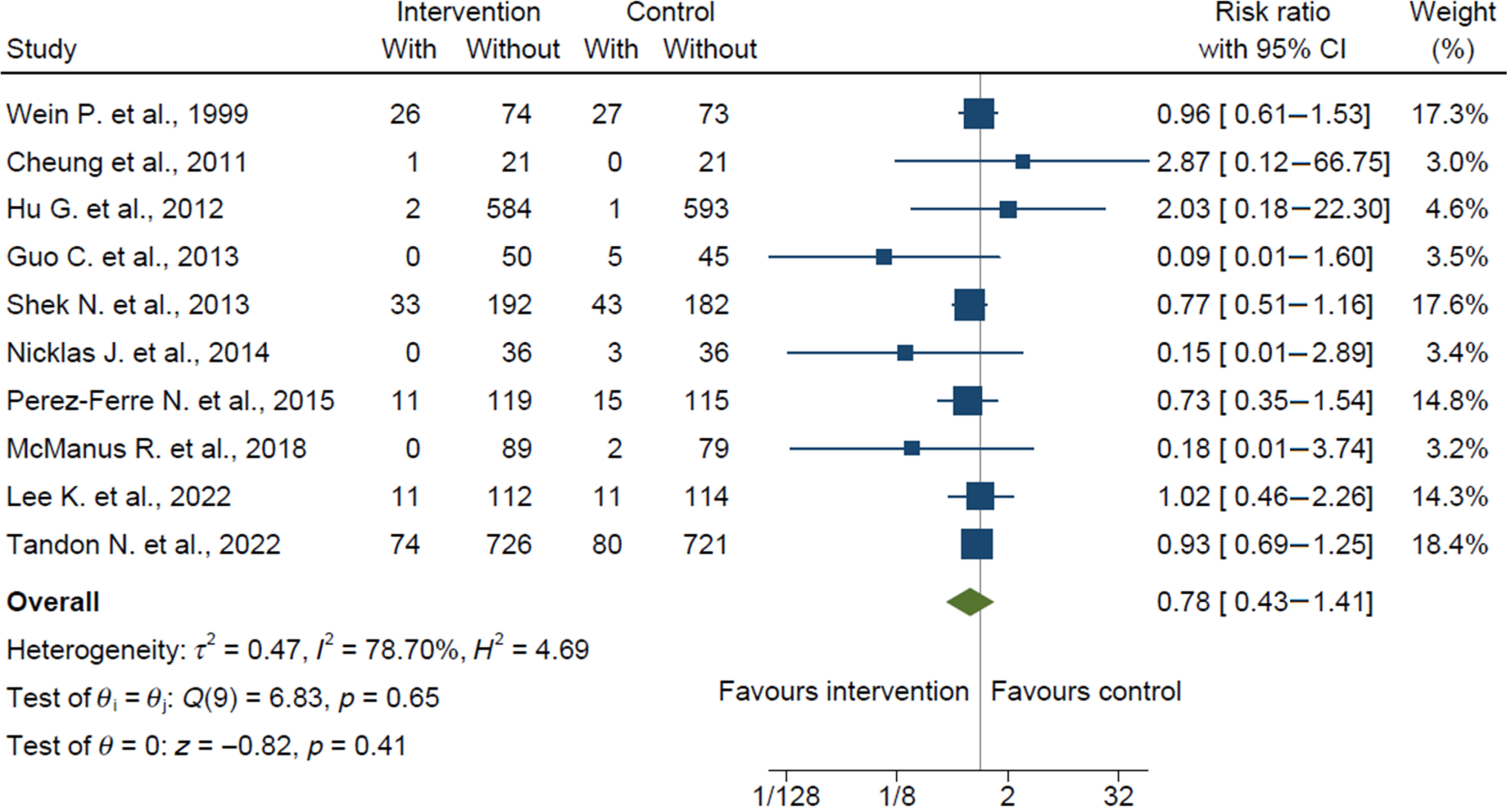
Primary outcome of the incidence of type 2 diabetes mellitus (risk ratio). CI, confidence interval.

**FIGURE 3:**
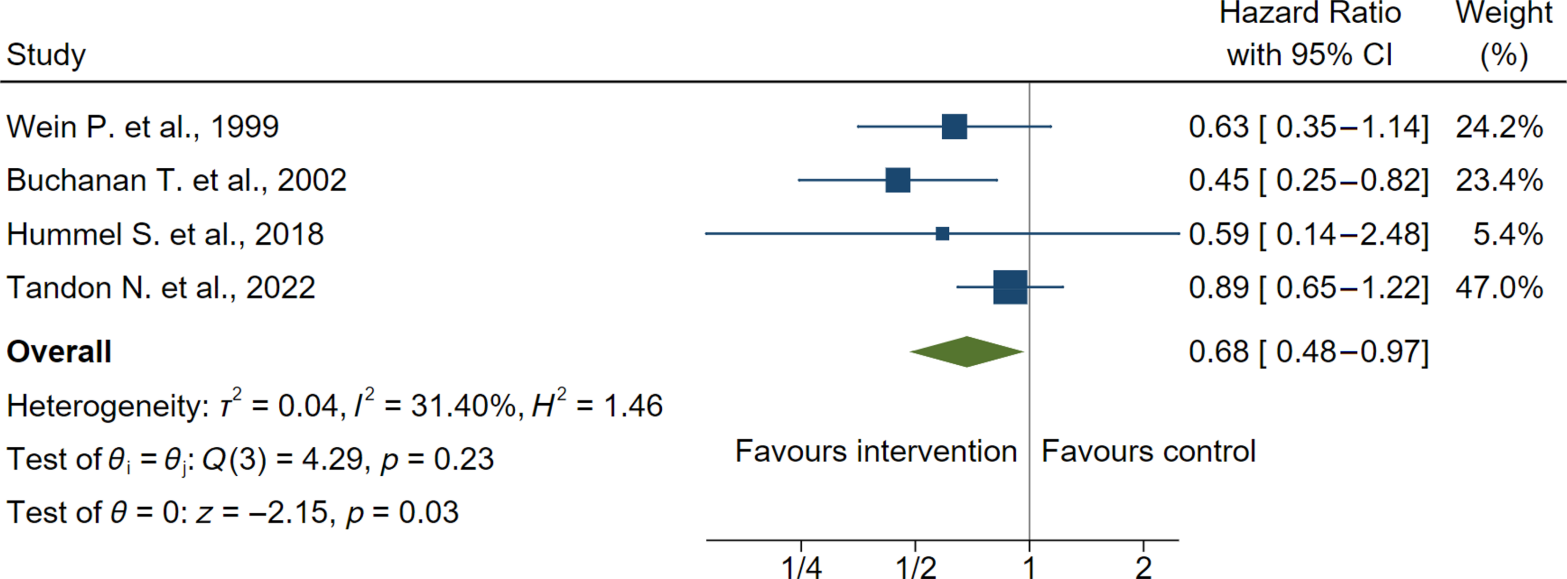
Primary outcome of the incidence of type 2 diabetes mellitus (hazard ratio). CI, confidence interval.

The pooled effects of the intervention, compared with control, on all outcomes are summarized in Table 2. Intervention was not associated with a significant reduction in the primary outcome of T2DM, compared with control, when all data from 10 eligible studies were included (risk ratio, 0.78; 95% CI: 0.43–1.41; *p* = 0.41; *I* ^2^ = 79%; Figure 2). However, the incidence of T2DM was significantly reduced among the four studies that utilized survival analysis (hazard ratio, 0.68; 95% CI: 0.48–0.97; *p* = 0.03; *I* ^2^ = 31%; Figure 3). For the secondary outcomes, there were significant reductions in fasting plasma glucose (−0.22 mmol/L; 95% CI: −0.39 to −0.04; *p* = 0.01; *I* ^2^ = 94%) and fasting insulin levels (−3.36 μIU/mL; 95% CI: −6.31 to −0.41; *p* = 0.03; *I* ^2^ = 97%), while a borderline significance in HOMA‐IR scores (−0.56; 95% CI: −1.12 to −0.01; *p* = 0.05; *I* ^2^ = 96%) but no significant differences in 2‐h OGTT or HbA_1_c measures. There were no differences in any of the anthropometric measures (body weight, BMI, and waist circumference) between the randomized groups. Significant reduction in LDL cholesterol (−0.31 mmol/L; 95% CI: −0.61 to −0.01; *p* = 0.04; *I* ^2^ = 95%) was observed in the intervention group compared with control, and a borderline significance was observed in total cholesterol (−0.24 mmol/L; 95% CI: −0.48 to −0.01; *p* = 0.05; *I* ^2^ = 90%), but not for HDL cholesterol and triglycerides.

**TABLE 2:** Pooled estimates of intervention effects on outcomes.

	No. of studies	No. of participants	Pooled effect size (95% CI)	*p*‐Value	*I* ^2^, %
T2DM, risk ratio^25^, ^27^, ^32^, ^33^, ^34^, ^35^, ^36^, ^37^, ^38^, ^39^, ^40^	10	4327	0.78 (0.43 to 1.41)	0.41	79
T2DM, hazard ratio^28^, ^31^, ^33^, ^36^	4	2180	0.68 (0.48 to 0.97)	0.03	31
2‐h OGTT, mmol/L^26^, ^29^, ^33^, ^38^	4	2003	−0.30 (−0.73 to 0.13)	0.17	78
Fasting plasma glucose, mmol/L^20^, ^25^, ^26^, ^27^, ^29^, ^30^, ^31^, ^33^, ^35^ ^,^ ^36^, ^38^, ^39^, ^40^	10	4711	−0.22 (−0.39 to −0.04)	0.01	94
HbA_1_c, %^25^, ^27^, ^32^, ^36^, ^38^	5	3459	−0.02 (−0.12 to 0.07)	0.64	83
Body weight, kg^20^, ^25^, ^26^, ^29^, ^30^, ^32^, ^34^, ^36^, ^38^	9	4230	−0.48 (−2.43 to 1.46)	0.63	93
BMI, kg/m^19^, ^20^, ^26^, ^27^, ^30^, ^32^, ^33^, ^34^, ^35^, ^37^, ^38^	10	2437	−0.51 (−1.46 to 0.44)	0.29	81
Waist circumference, cm^20^, ^25^, ^26^, ^27^, ^29^, ^30^, ^32^, ^36^, ^38^	9	4415	−1.05 (−2.45 to 0.35)	0.14	66
HOMA‐IR^19^, ^20^, ^26^, ^27^, ^30^, ^38^	6	1949	−0.56 (−1.12 to −0.01)	0.05	96
Fasting insulin, μIU/mL^20^, ^27^, ^38^	3	1620	−3.36 (−6.31 to −0.41)	0.03	97
Total cholesterol, mmol/L^20^, ^27^ ^,^ ^33^, ^34^, ^35^, ^36^, ^38^, ^40^	8	2757	−0.24 (−0.48 to −0.01)	0.05	90
HDL cholesterol, mmol/L^20^, ^25^, ^26^, ^27^, ^28^, ^29^, ^30^, ^38^	8	2757	0.03 (−0.08 to 0.13)	0.65	95
LDL cholesterol, mmol/L^20^, ^25^, ^26^, ^27^, ^28^, ^29^, ^30^, ^38^	8	2757	−0.31 (−0.61 to −0.01)	0.04	95
Triglycerides, mmol/L^20^, ^25^, ^26^, ^27^, ^28^, ^29^, ^30^ ^,^ ^35^, ^36^, ^38^, ^40^	8	2757	−0.02 (−0.19 to 0.16)	0.87	93

Abbreviations: BMI, body mass index; CI, confidence interval; HDL, high‐density lipoprotein; HOMA‐IR, Homeostatic Model Assessment for Insulin Resistance; LDL, Low‐density lipoprotein; OGTT, oral glucose tolerance test; HbA_1_c, Haemoglobin A1c; T2DM, type 2 diabetes mellitus.

### 3.4 Subgroup analyses

**FIGURE 4:**
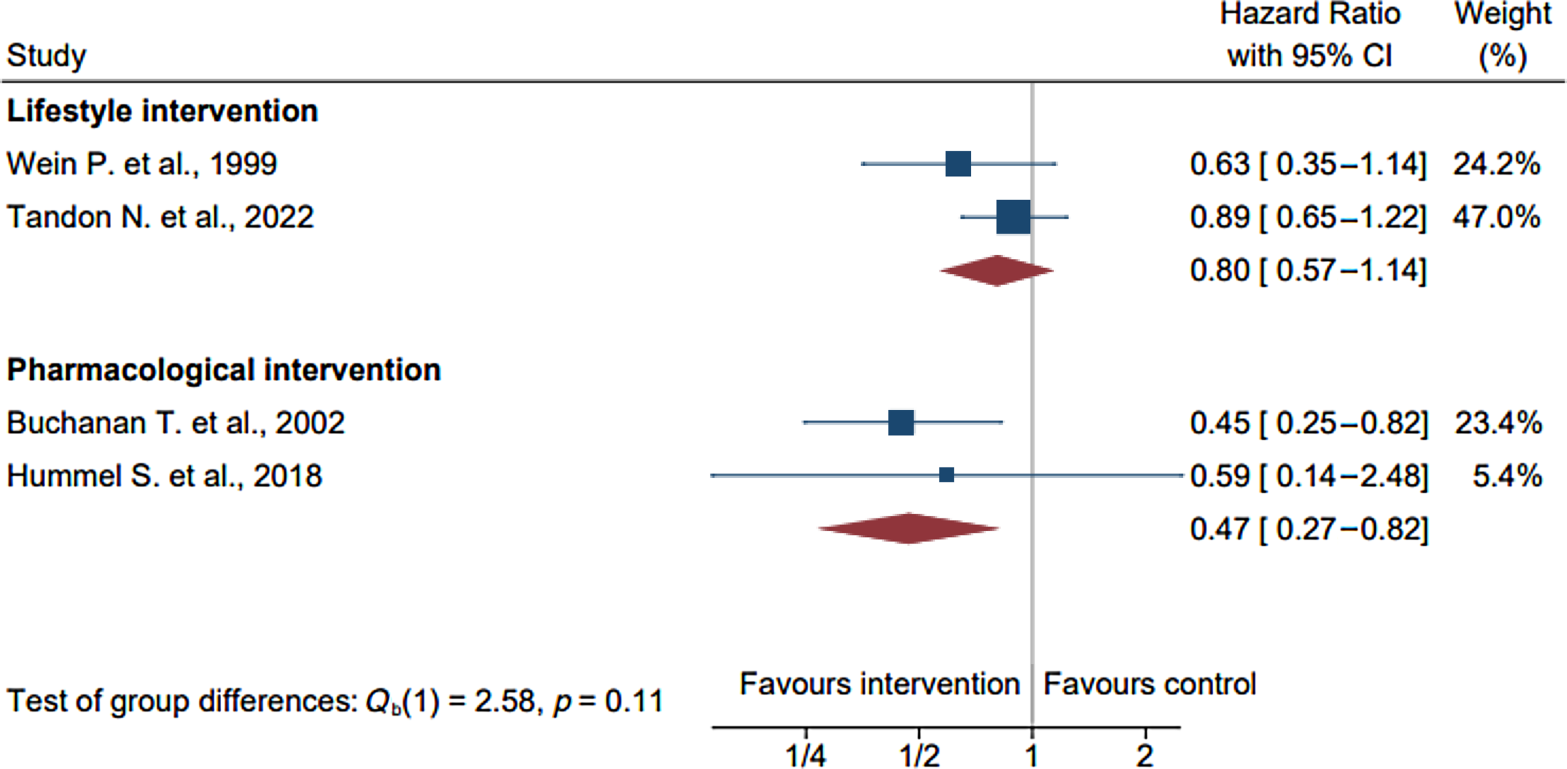
Subgroup analysis by type of intervention. CI, confidence interval.

Subgroup analysis by the type of intervention, baseline BMI, and country income class are summarized in Table 3. There was no significant heterogeneity between subgroups, despite the effect size estimates potentially favoring greater benefit with pharmacological (*n* = 379; hazard ratio, 0.47; 95% CI: 0.27–0.82; *I* ^2^ = 1%) compared with lifestyle interventions (*n* = 1801; hazard ratio, 0.8; 95% CI: 0.57–1.14; *I* ^2^ = 25%; *p* = 0.11; Figure 4). No significant heterogeneity were observed among studies on participants with obesity (*n* = 2094; hazard ratio, 0.74; 95% CI: 0.30–1.80; *I* ^2^ = 62%) compared with studies on participants without obesity (*n* = 2133; hazard ratio, 0.87; 95% CI: 0.57–1.33; *I* ^2^ = 37%), and in studies conducted in high‐income countries (*n* = 748; hazard ratio, 0.71; 95% CI: 0.27–1.84; *I* ^2^ = 61%) compared with those conducted in middle‐income countries (*n* = 3579; hazard ratio, 0.83; 95% CI: 0.37–1.84; *I* ^2^ = 83%).

**TABLE 3:** Subgroup analysis on the incidence of type 2 diabetes mellitus (T2DM).

	No. of studies	No. participants	Pooled effect size (95% CI)	*I* ^2^	*p*‐Value for test of subgroup difference
T2DM, risk ratio^25^, ^27^, ^32^, ^33^, ^34^, ^35^, ^36^, ^37^, ^38^, ^39^	10	4327	0.78 (0.43–1.41)	79	‐
Subgroup analyses for T2DM, risk ratio					
By type of intervention					
Lifestyle^25^, ^27^, ^32^, ^33^, ^34^, ^35^, ^36^, ^37^, ^38^, ^39^	10	4327	0.78 (0.43–1.41)	79	‐
By baseline BMI^a^					
Obese^25^, ^32^, ^34^, ^36^	4	2094	0.74 (0.30–1.80)	62	0.30
Nonobese^27^, ^33^, ^35^, ^37^, ^38^	5	2133	0.87 (0.57–1.33)	37	
Not reported^39^	1	100	0.09 (0.01–1.60)	‐	
By country of study's income class					
High‐income^27^, ^32^, ^33^, ^34^, ^35^	5	748	0.71 (0.27–1.84)	61	0.81
Middle‐income^25^, ^36^, ^37^, ^38^, ^39^	5	3579	0.83 (0.37–1.84)	83	
T2DM, hazard ratio^28^, ^31^, ^33^, ^36^	4	2180	0.68 (0.48–0.97)	31	‐
Subgroup analyses for T2DM, hazard ratio					
By type of intervention					
Lifestyle^33^, ^36^	2	1801	0.80 (0.57–1.14)	25	0.11
Pharmacological^28^, ^31^	2	379	0.47 (0.27–0.82)	1	
By baseline BMI					
Obese^31^, ^35^	2	1867	0.67 (0.35–1.26)	72	0.88
Nonobese^28^, ^33^, ^36^	2	313	0.63 (0.37–1.08)	0	
By country of study's income class					
High income^28^, ^31^, ^33^	3	579	0.54 (0.36–0.82)	4	0.06
Middle income^36^	1	1601	0.89 (0.65–1.22)	‐	

Abbreviation: CI, confidence interval.

^a^ Categorized according to race specific body mass index (BMI).

### 3.5 Sensitivity analysis

The sensitivity analysis excluding high risk of bias studies and the suspected outlier did not change the effect observed in most outcomes, apart from HOMA‐IR and LDL cholesterol. Removal of Zilberman et al.,^20^ identified as the outlier and having a high risk of bias, shifted differences in HOMA‐IR from being not significant (−0.56; 95% CI: −1.12 to −0.01; *p* = 0.05; *I* ^2^ = 96%) to significant (−0.38; 95% CI: −0.58 to −0.18; *p* < 0.01; *I* ^2^ = 62%), and the change in LDL cholesterol from being significant (−0.31; 95% CI: −0.61 to −0.01; *p* = 0.04; *I* ^2^ = 95%) to not significant (−0.19; 95% CI: −0.67 to 0.01; *p* = 0.06; *I* ^2^ = 86%).

## 4 DISCUSSION

The current review suggests equivocal findings with respect to the effects of interventions aimed at preventing T2DM among women with a recent history of GDM. Overall, there was substantial heterogeneity between trials. Statistically significant benefits were identified only in a meta‐analysis of four trials where survival analysis was used, with a wide 95% CI. Point estimates suggested that the benefits of pharmacological intervention may be greater than that of lifestyle strategies; however, the difference was not statistically significant. The limited number of pharmacotherapy studies highlights the need for further trials, of which three is currently underway.^12^, ^13^, ^40^ The current living systematic review planned annually for the next 5 years will provide up‐to‐date evidence of the effects as they emerge.

This is the first review to include any type of intervention to reduce the risk of T2DM in women post‐GDM. While our analyses suggest the possibility of a greater benefit with pharmacological compared with lifestyle interventions, the small number of studies limits our ability to draw firm conclusions. Similarly, the most recent scoping review of pharmacological interventions in this population highlighted the need for further pharmacological studies.^11^ One pharmacological study included in this review was terminated prematurely due to hepatotoxicity concerns and the drug, troglitazone, has been withdrawn for use.^23^ Therefore, only two trials to date are of clinical relevance. A search of the Clinical Trial Registries shows three studies^12^, ^13^, ^40^ in progress that are evaluating the effects of liraglutide and semaglutide on incidence of T2DM in women post‐GDM. While the results of these studies will provide further evidence on the effects of pharmacological interventions, the relatively small sample sizes (*n* = 80–206) indicate a need for large robust trials to provide definitive data.

Approaches to modify lifestyle intervention were the most common intervention type evaluated, and we found equivocal evidence that these interventions reduced the risk of T2DM in women with a history of GDM. Efficacy of lifestyle intervention depends heavily on adherence to intervention, and several factors are found to influence this including having reputable intervention facilitators, providing personalized guidance, and supervised sessions.^41^ While interventions in all but one study were provided by reputable intervention facilitators, only 50% of the physical activity and 61% of diet interventions were personalized, with only two studies providing supervised sessions. Furthermore, only two studies reported adherence to intervention, which was relatively low with reports of 34% attending at least one walking session and 58.3% attending 50% or more of the provided sessions. Although retention rate for most of the studies were high, adherence to intervention is unknown, which is of concern as adherence to intervention is especially problematic in this population.^42^ Therefore, reporting of adherence to intervention components (to the extent feasible) is strongly recommended in future intervention studies in postpartum women.

Unlike the current review, a recently published lifestyle interventions post‐GDM found interventions to be associated with significant reductions in the risk of developing T2DM.^10^ This review included intervention delivered at any time following pregnancy affected by GDM while we focused only on interventions delivered relatively early in the postpartum period. We thus excluded the subgroup analysis of the Diabetes Prevention Program^43^ where both lifestyle and metformin were found to be beneficial in a subset of women (*n* = 350) with a history of GDM (average 656 weeks postpartum).^43^ It is uncertain whether such benefits observed in an older cohort more distant to their index pregnancy would extend to interventions delivered in the early postpartum period. Furthermore, our review only included studies with at least 12 months follow‐up to reduce the likelihood of chance findings. For example, the review by Ratnakaran et al.^10^ included a study with a duration of 4 months, which showed a significant benefit with the lifestyle intervention.^44^ Therefore, we believe the current review that focuses on intervention delivered early postpartum, and with a follow‐up of at least 12 months in duration, report findings that are robust and specific to women with a recent history of GDM.^45^ Furthermore, this may explain why the current review found no significant improvements in anthropometric measures but did in fasting glucose and insulin levels different to other systematic reviews.^45^, ^46^ This is also the first review to analyze changes to lipid profiles and to show reductions in LDL cholesterol. However, this shifted significance when a sensitivity analysis was conducted by excluding a study with high‐risk of bias. The sensitivity analysis also shifted the significance of HOMA‐IR from not significant to significant, suggesting improvements in the intervention group compared with controls. These benefits would be favorable long‐term; however, the current review with a median follow‐up of 24 months may not have been long enough to capture this translation into reductions in T2DM. Therefore, although the evidence of the effects suggests a benefit, the small number of trials and participants and the short follow‐up period limit our ability to make firm conclusions.

The current review specified clear criteria to try and identify effectiveness of preventive interventions, of at least 12 months in duration, on reducing the risk of T2DM in women with a recent history of GDM. While this allowed us to answer our proposed question, it limited the number of included studies. Furthermore, combining different types of intervention resulted in large heterogeneity of the outcomes. However, by combining the different intervention types, the current review identified the need for more adequately powered pharmacotherapy interventions to examine its efficacy.

## 5 CONCLUSION

This review indicates that the benefits of preventive interventions to reduce the risk of T2DM in women post‐GDM when initiated within 5 years postpartum remain equivocal. While there is little debate that improving diet and physical activity, and other changes to lifestyle, can prevent T2DM, sustainable and scalable strategies to effectively achieve such changes in this population remain uncertain. Pharmacological approaches show promise, but the evidence is sparse. This living systematic review for the next 5 years will provide updated evidence on preventive interventions.

## AUTHOR CONTRIBUTIONS

VL, AP, YG, and NT conceived and designed the study. VL and MM conducted the screening, identification, and extraction of data. RL provided translation support and extracted data for the papers published in Chinese. GD and JAS performed the analysis. VL drafted the initial manuscript. AG, SA, DP, JL, and HD contributed to manuscript revisions, discussions, and findings.

## FUNDING INFORMATION

Anushka Patel is supported by an National Health and Medical Research Council Investigator Grant Investigator Grant (APP2016801).

## CONFLICT OF INTEREST STATEMENT

Anushka Patel is an Editorial Board member of Journal of Diabetes and a co‐author of this article. To minimize bias, she was excluded from all editorial decision‐making related to the acceptance of this article for publication.

## Supporting information


**Data S1.** Supplementary Appendix.
