# Supplementary material for: The efficacy of interventions to prevent type 2 diabetes among women with recent gestational diabetes mellitus—A living systematic review and meta‐analysis
Source: J Diabetes. 2024 Aug 13;16(8):e13590. doi: 10.1111/1753-0407.13590 (PMC11320752; doi:10.1111/1753-0407.13590)
Supplement: Supplementary file 1 — Data S1. Supplementary Appendix. [file JDB-16-e13590-s001.docx]

**Supplementary Appendix 1.**

List of extracted variables

- Study characteristics
  - Title
  - Author, Year
  - Journal
  - Language
  - Type of Paper
  - Recruitment Start Year
  - Recruitment End Year
  - Intervention Period (Months)
  - Follow-up Period (months)
  - Sample Size
  - Allocated sample size
  - Trial Design
  - Single/Multi-Centre
  - GDM criteria used
  - Inclusion Criteria
  - Exclusion Criteria
  - Intervention postpartum/during pregnancy
  - Weeks postpartum intervention commenced
- Intervention
  - Intervention groups
  - Intervention
  - Mode of delivery
  - Group or individual
  - Session design
  - Duration of each session (min)
  - Number of sessions
  - Total number of hours
  - Intervention delivered by
  - Goal of intervention
- Participant baseline characteristics
  - Country
  - Actual sample size
  - Age
  - Weight (kg)
  - BMI (kg/m^2^)
  - Waist Circumference (cm)
  - Hypertensive participants
  - Ethnicity
  - Insulin use during pregnancy
- Outcomes
  - Actual final sample size
  - Body Weight (kg)
  - BMI (kg/m^2^)
  - Waist Circumference (cm)
  - Incidence of T2DM
  - Incidence of pre-diabetes
  - 2-hour OGTT
  - HbA1C
  - HOMA-IR
  - Fasting BGL
  - Fasting Insulin
  - Total cholesterol
  - HDL-Cholesterol
  - LDL-Cholesterol
  - Triglyceride

Appendix 2. TIDier checklist

|  | **Why** | **What** | |  |  |  |  |  |  |  |  | **Who provided** | **How** | |  |  |
| --- | --- | --- | --- | --- | --- | --- | --- | --- | --- | --- | --- | --- | --- | --- | --- | --- |
|  |  |  |  | **Physical activity** | | | | | | **Diet** | **Control** |  |  |  |  |  |
| **Author** | **Goal of INT** | **INT type** | **INT duration (months)** | **Individual**  **tailoring** | **Supervised** | **Type of exercise** | **Frequency (per week)** | **Duration (week)** | **Duration of each (min)** | **Individual**  **tailoring** | **INT** | **INT facilitator** | **Delivery mode** | **Group/individual** | **ADH** | **RET** |
| Cheung et al., 2011 | (1) 30 min moderate-intensity activity on 5 days (150 min/week) or 10,000 steps/day 5 days/week at 1 year. | PA | 12 | Y | N | x | x | x | x | x | Education | Counsellor | F+T | I | x | 79.1% |
| Guo et al., 2013 | (1) Prevent diabetes and depression (2) Improve health awareness and blood sugar control  (3) Diet: control total energy intake per day (weight in kg times 20 - 40 kCal) | PA+D | 12 | N | N | x | x | x | x | Y | Usual care | Physicians, nurses, and dietician | F+T | I | x | 100% |
| Hu et al., 2012 | (1) Reduction in 5-10% initial body weight if BMI ≥24 kg/m² but no weight loss if BMI < 24 kg/m² (2) Diet target: total fat intake <30% of energy consumed, saturated fat intake <10% of energy consumed, carbohydrate intake 55-65% of energy consumed, and fibre intake 20-30g/day (3) Moderate or vigorous exercise for at least 30 mins daily | PA+D | 12 | Y | N | x | x | x | x | Y | Education | Dietitian | F+T | I | x | 91% |
| Lee et al., 2022 | (1) Adherence to diabetes screening  (2) Lifestyle modification (breastfeeding, diet and exercise) | PA+D | 24 | Y | N | x | x | x | x | Y | Usual care | Nurse, physician, physiotherapist and dietitian | F | I | x | 45.8% |
| McManus et al., 2018 | (1) Achieve 7% weight reduction  (2) Healthy diet: breastfeeding for baby and maternal health; increasing intake of whole grains, vegetables, fruits, and high-fibre cereals/snacks; eating meals as a family (3) Physical activity: 30 minutes of intentional muscle activity most days of the week | PA+D | 12 | N | Y | Aerobic | 1 | 52 | 60 | N | Education | Study coordinator | F+T+W | B | 34% attended weekly walking group at least once.  67.4% accessed the study website at least once. | 57.1% |
| Nicklas et al., 2014 | (1) Return to pre-pregnancy weight (2) Emphasized dietary choices that would transition readily from the pregnancy GDM diet, including lower glycaemic index, higher fibre, and controlled portion sizes (3) Gradually increase physical activity to ≥150 min/week, including resistance training. | PA+D | 12 | Y | N | x | x | x | x | Y | Education | Dietitian | W | I | x | 90.7% |
| O'Dea A et al., 2015 | Health lifestyle change: (1) Improving quality and enjoyment of life  (2) Management of blood pressure, lipids, glucose and adherence with cardio-protective medications | PA+D | 12 | Y | Y | x | 1 | 12 | 60 | Y | Education | Nurses, dieticians, physical activity specialists and physician | F | B | 58.3% attended ≥6 sessions out of 12 | 72.0% |
| O'Reilly et al., 2016 | (1) Diet changes to <30% energy from fat, <10% energy from saturated fat, > 15g dietary fibre per 1,000kcal (2) >30 min moderate physical activity at least 5 days per week (3) >5% body weight reduction | PA+D | 12 | N | N | x | x | x | x | N | Usual care | Specially trained healthcare professionals | F+T | B | x | 75.7% |
| Perez-Ferre et al, 2015 | (1) Adhere to Mediterranean diet pattern (Achieve a nutrition score greater than 5 based on the previously reported Diabetes Nutrition and Complications Trial) (2) Aerobic exercise for at least 150 minute per week | PA+D | 36 | Y | Y^ | Aerobic and resistance | 4 | 10 | 50-60 | Y | Education | Physiotherapists, doctors, nurses, dietitians and endocrinologists | F+T | B | x | 91.2% |
| Shek et al., 2013 | (1) Prevent diabetes (2) Optimal caloric intake (Harris–Benedict’s equation) | PA+D | 36 | Y | N | x | x | x | x | Y | Usual care | Dietitian and research nurses | F | I | x | 74.7% |
| Tandon et al., 2022 | (1) Prevent weight gain (Achieve and maintain weight reduction goals) by limiting fat (especially saturated fat), glucose and sodium intake and increase fibre intake. (2) Engage in physical activity for at least 30 minutes a day. | PA+D | 12 | N | N | x | x | x | x | N | Usual Care | Counsellors, nurses and nurse auxiliaries | F+T | G* | x | 89.1% |
| Wein et al., 1999 | (1) Follow a healthy diet  (2) Increase physical activity | PA+D | 51 | N | N | x | x | x | x | N | Education | Dietitian | T | I | x | 96.5% |
| Yu et al., 2012 | (1) Improve insulin resistance and/or function of β cell | PA+D | 24 | N | N | x | x | x | x | Y | Education | Physicians, nurses, and dietician | T | I | x | 100% |
| Zilberman-Kravits et al., 2018 | (1) Adhere to a healthy lifestyle (2) Increase physical activity to > 150 min/week | PA+D | 24 | N | N | x | x | x | x | N | Education | Nurse, Dietitian, and sports instructors | F | B | x | 57.8% |

INT = intervention, ADH = adherence, RET = retention, ^= partial supervision, * = Some participants provided individual sessions if aim not achieved, PA: Physical activity, D: Diet x=n/a, Y= Yes, N= No, F= Face to face, T= telephone, W= web based, I= individual, B= Both group and Individual

**
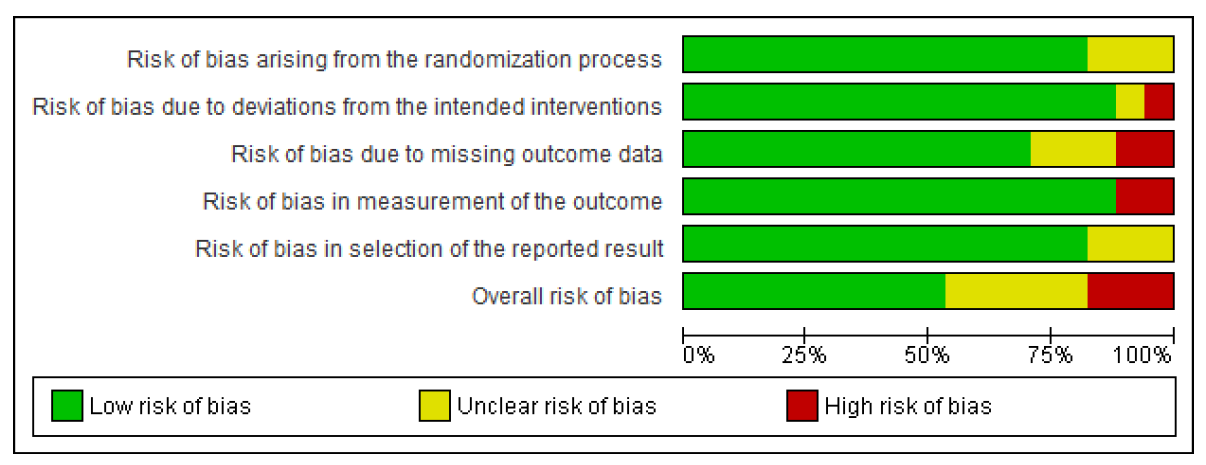
**

**
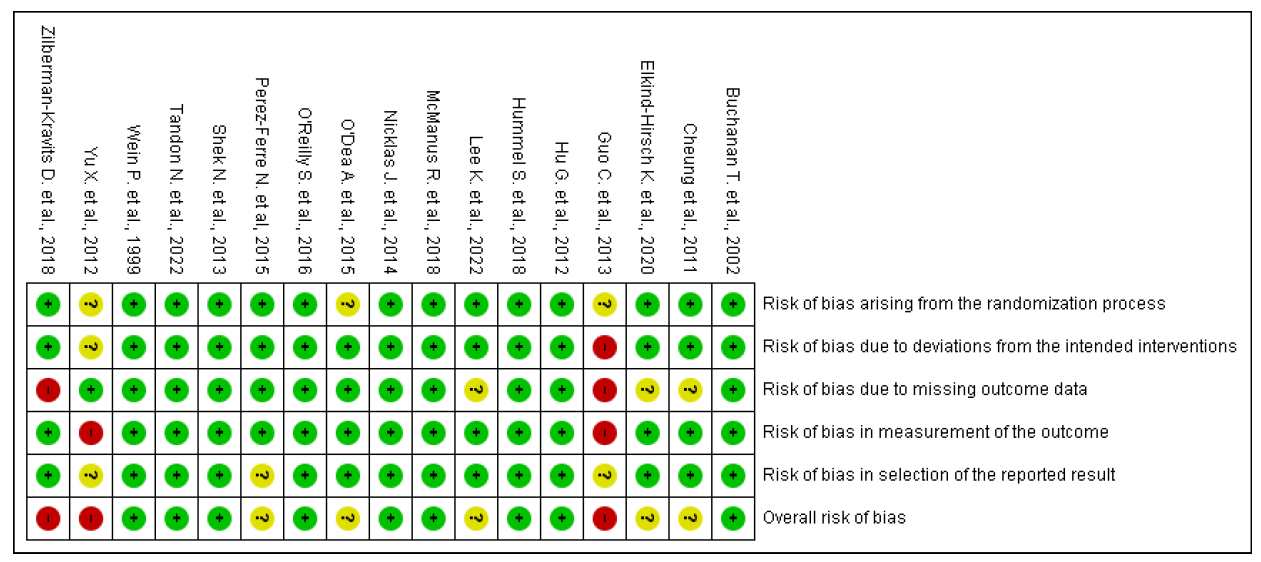
**

**Supplementary Figure 1.** Cochrane Risk-of-bias summary.


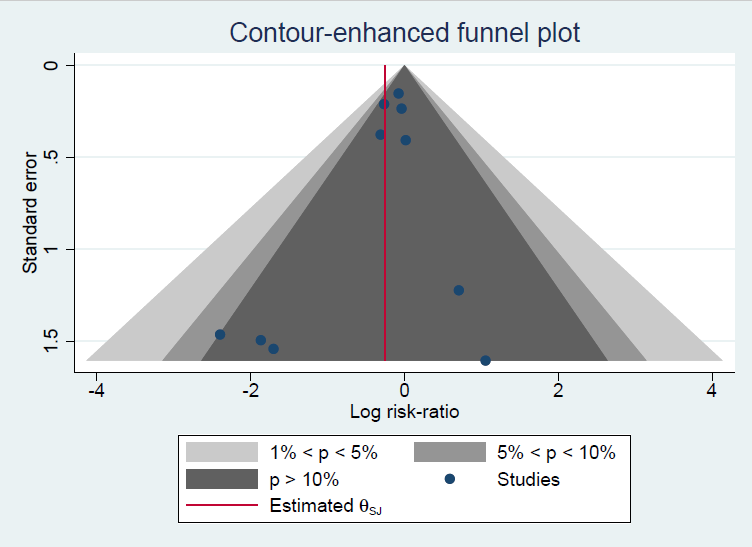


**Supplementary Figure 2.** Funnel plot of studies reporting incidence of Type 2 Diabetes Mellitus.
